# Integrating Gold Nanoparticles with Brachytherapy: In Vitro Insights into Radiosensitization in Cervical Cancer

**DOI:** 10.3390/cancers18183025

**Published:** 2026-09-17

**Authors:** Maria Anthi Kouri, Maria-Eleni Kalkou, Kalliopi Platoni, Nikos Kollaros, Kyveli Zourari, Marina Chalkia, George Patatoukas, Aris Spathis, Vassilis Kouloulias, Efstathios Efstathopoulos

**Affiliations:** 1 Department of Applied Medical Physics, Medical School, Attikon University Hospital, National and Kapodistrian University of Athens, 11527 Athens, Greece; mariakouri90@gmail.com (M.A.K.); marilou94kal@gmail.com (M.-E.K.); polaplatoni@gmail.com (K.P.); nkollaros08@gmail.com (N.K.); kzourari@med.uoa.gr (K.Z.); chalkiamar@outlook.com (M.C.); gpatatouk@gmail.com (G.P.); 2 Radiation Physics Materials Technology and Biomedical Imaging Laboratory (AΚΤΥBA), Department of Biomedical Engineering, University of West Attica, Egaleo, 12210 Athens, Greece; 3 Medical Physics, General Hospital GHA Korgialeneio Mpenakeio-Hellenic Red Cross, 11526 Athens, Greece; 4 2nd Department of Pathology, University General Hospital “ATTIKON”, National and Kapodistrian University of Athens, 12462 Athens, Greece; arisspa@gmail.com; 5 Departement of Radiation Oncology, Medical School, Attikon University Hospital, National and Kapodistrian University of Athens, 11527 Athens, Greece; vkouloul@med.uoa.gr

**Keywords:** gold nanoparticles, brachytherapy, SiHa cancer cell line, apoptosis, dose enhancement, radiosensitization, radiobiology, linear quadratic model

## Abstract

**Background/Objectives:** Cervical cancer treatment relies heavily on high-dose-rate (HDR) ^192^Ir brachytherapy; however, therapeutic efficacy remains limited by tumor radioresistance and the inability to escalate dose without increasing toxicity to surrounding healthy tissues. The present study investigates the potential of gold nanoparticles (AuNPs) to enhance radiosensitivity under clinically relevant ^192^Ir brachytherapy conditions through the combined action of physical dose amplification and radiobiological modulation. Particular emphasis is placed on the unique radiophysical interactions generated by the mixed gamma and secondary beta emissions of ^192^Ir, which, in the presence of high atomic number nanoparticles, promote localized photoelectric absorption and the emission of low-range secondary electrons, including Auger electrons. The study further aims to determine how AuNPs size and post-irradiation temporal evolution influence radiation-induced cytotoxicity and apoptosis in cervical cancer cell lines, thereby providing a biologically representative model of nanoparticle-assisted brachytherapy. **Methods:** Clonogenic survival, dose enhancement factor (DEF), and apoptosis were evaluated following irradiation in the presence of 10 nm and 50 nm AuNPs in two independent biological experiments (*n* = 2). **Results:** A clear dose-dependent reduction in survival fraction and increase in apoptosis were observed in nanoparticle-treated groups compared with irradiation alone. Radiosensitization demonstrated strong size dependence, with 50 nm AuNPs producing the greatest enhancement, a finding that may reflect size-dependent differences in cellular internalization, intracellular distribution, and nanoscale energy deposition previously established in AuNP studies. Importantly, biological effects intensified at later post-irradiation intervals, demonstrating a sustained temporal evolution of the radiobiological response beyond the initial irradiation event, potentially involving oxidative and other delayed cellular stress mechanisms described in AuNP radiosensitization. **Conclusions:** These findings demonstrate the capacity of AuNPs to enhance the radiobiological response of cervical cancer cells to ^192^Ir brachytherapy and identify nanoparticle size and post-irradiation time as important determinants of this effect. They therefore support AuNP-assisted brachytherapy as a promising strategy for further development toward biologically optimized radiotherapy capable of improving tumor response without escalation of the prescribed radiation dose.

## Simple Summary

Brachytherapy is a key component of cervical cancer treatment, delivering highly localized radiation doses to the tumor while limiting exposure to surrounding healthy tissues. Gold nanoparticles (AuNPs) have emerged as promising radiosensitizing agents, with their interaction with ionizing radiation capable of modifying local energy deposition and enhancing subsequent radiobiological effects. This study evaluated the influence of gold nanoparticle size on the radiosensitization of cervical cancer cells exposed to high-dose-rate (HDR) ^192^Ir brachytherapy. AuNPs of 10 and 50 nm were investigated across different radiation doses and post-irradiation time points, using clonogenic survival and apoptosis as radiobiological endpoints. AuNPs treatment reduced clonogenic survival and increased apoptosis, with 50 nm nanoparticles producing greater radiosensitization than 10 nm nanoparticles under the investigated conditions. These findings provide preliminary in vitro evidence of size-dependent radiosensitization under iridium-192 brachytherapy and highlight the relevance of AuNPs characteristics in the development of nanoparticle-assisted radiotherapy strategies.

## 1. Introduction

Cervical cancer remains a major global health challenge, with radiotherapy playing a central role in its management [1,2]. In particular, high-dose-rate (HDR) brachytherapy is an essential component of definitive treatment, enabling the delivery of highly conformal radiation doses directly within or adjacent to the tumor [3,4]. The use of iridium-192 (^192^Ir) sources allows for steep dose gradients and high tumor doses while minimizing exposure to surrounding healthy tissues, which is critical for improving local control and reducing toxicity to organs at risk [5,6]. However, treatment efficacy is often limited by intrinsic or acquired radioresistance, as well as by the need to constrain dose escalation due to normal tissue tolerance. Consequently, strategies that can enhance tumor radiosensitivity without increasing the prescribed dose are of significant clinical interest [7,8,9].

From a radiophysical perspective, ^192^Ir emits a broad photon energy spectrum with a mean energy of approximately 380 keV, where Compton scattering dominates energy deposition in soft tissue. Nevertheless, the introduction of high atomic number (high-Z) materials such as gold can locally modify radiation interactions [8,10,11]. Gold nanoparticles (AuNPs) enhance the probability of photoelectric absorption in their immediate vicinity, resulting in increased emission of low-energy secondary electrons, including Auger and photoelectrons [10,12,13]. Due to their short range, these electrons deposit energy over nanometric distances, leading to highly localized dose amplification at the subcellular level [14]. This nanoscale heterogeneity in energy deposition is particularly relevant in brachytherapy, where the proximity of the radiation source and the spatial distribution of dose are critical determinants of biological effect [15,16].

Beyond physical dose enhancement, AuNPs also modulate biological responses to radiation. Numerous studies have demonstrated that AuNPs increase the production of reactive oxygen species (ROS), promote DNA double-strand break formation, and interfere with DNA repair pathways, thereby enhancing radiation-induced cell death [17,18]. Importantly, these effects are not limited to immediate damage but extend to delayed processes, including oxidative stress, mitochondrial dysfunction, and apoptosis [19,20]. The extent of these effects is strongly influenced by nanoparticle characteristics, particularly size, which governs cellular uptake, intracellular distribution, and interaction with critical targets such as the nucleus and mitochondria. In mammalian cells, nanoparticles in the range of ~50 nm have been shown to exhibit optimal internalization, potentially leading to greater radiosensitization compared to smaller particles [21,22].

While AuNP-mediated radiosensitization has been extensively investigated under megavoltage external beam irradiation, relatively limited data exist for clinically relevant brachytherapy conditions [23]. In addition, the temporal dynamics of radiation-induced responses, including delayed apoptosis and long-term clonogenic survival, remain incompletely characterized in nanoparticle-treated systems. Given the distinct photon energy spectrum of ^192^Ir and the importance of brachytherapy in cervical cancer treatment, it is essential to evaluate nanoparticle effects within this specific radiobiological context [24,25,26].

The scope of the present study is to investigate the synergistic integration of high-dose-rate brachytherapy with gold nanoparticle (AuNP)–mediated radiosensitization under clinically relevant conditions for cervical cancer [27,28]. Specifically, the study investigates whether the interaction between the complex photon energy spectrum of ^192^Ir and the radiophysical properties of high-Z AuNPs is translated into measurable differences in radiobiological response according to nanoparticle size, radiation dose, and post-irradiation time. AuNPs of 10 and 50 nm were selected to compare two distinct particle sizes with different size-dependent cellular and radiophysical behavior, with ~50 nm particles previously associated with particularly efficient cellular internalization [22]. The selected concentration of 5 μg/mL allowed the radiosensitizing response of the two AuNP sizes to be investigated under the same nanoparticle exposure conditions. Radiation doses of 1.5, 3.0, and 4.5 Gy were selected to evaluate the progressive dose-dependent response under ^192^Ir HDR irradiation, while assessment at 0, 24, and 48 h enabled characterization of the temporal evolution of the radiation-induced response, particularly apoptosis [24]. Accordingly, the study hypothesis was that AuNP-mediated radiosensitization under ^192^Ir HDR brachytherapy would depend on nanoparticle size and would be reflected in differences in clonogenic survival, dose enhancement, and apoptosis across radiation dose and post-irradiation time.

## 2. Materials and Methods

### 2.1. Cell Culturing Protocol

The human cervical squamous cell carcinoma cell line SiHa (HTB-35) was obtained from the American Type Culture Collection (ATCC, Manassas, VA, USA) [29]. Cells were cultured in accordance with the supplier’s instructions at the Clinical Biochemistry Laboratory, Attikon General Hospital, Medical School, National and Kapodistrian University of Athens, Athens, Greece. SiHa cells were maintained in Dulbecco’s Modified Eagle Medium (DMEM) supplemented with 1 g/L glucose, sodium pyruvate, 10% fetal bovine serum (FBS), 1% penicillin–streptomycin, amphotericin B, non-essential amino acids, and L-glutamine. Cultures were incubated at 37 °C in a humidified atmosphere containing 5% CO_2_. Cells were subcultured upon reaching approximately 70–80% confluency. For passaging, the culture medium was removed and the cell monolayer was rinsed with 1× PBS (Capricorn Scientific, Hesse, Germany) to remove residual serum. Cells were then incubated with 0.25% (*w*/*v*) trypsin–0.53 mM EDTA for 2–5 min at 37 °C until detachment was observed. Enzymatic activity was neutralized with complete medium, and cells were gently resuspended. Cells were seeded into 24-well flat-bottom plates at a density of 1.15 × 10^5^ cells per well in 1 mL of complete growth medium. Cell viability and counting were determined using a hemocytometer and Trypan Blue exclusion assay.

### 2.2. Gold Nanoparticle (AuNPs) Treatment

Gold nanospheres (AuNPs) with nominal diameters of 10 nm and 50 nm were purchased from nanoComposix (San Diego, CA, USA). The particles were BioPure™ Gold Nanospheres, PEG, with product numbers AUGB10 and AUGB50-1M, respectively. The 10 nm particles (AUGB10; Lot No. SXV0034) had a TEM diameter of 11.4 ± 0.9 nm, a hydrodynamic diameter of 36 nm, and a zeta potential of −10 mV, according to the manufacturer’s Certificate of Analysis. The particles were functionalized with mPEG (5 kDa) and supplied in USP purified water at a gold concentration of 1.05 mg/mL. The 50 nm particles (AUGB50-1M) were supplied as a PEG-functionalized gold nanosphere suspension at a nominal concentration of 1 mg/mL in water. A concentration of 5 μg/mL was selected for both gold nanoparticle formulations based on our previous experimental work, which supported the use of this concentration under the experimental conditions investigated. Accordingly, 5 μg/mL was used throughout the present study for both the 10 nm and 50 nm gold nanoparticles. Cells were incubated with the nanoparticles for 24 h prior to irradiation, consistent with the exposure period used in previous studies investigating nanoparticle-mediated radiosensitization.

### 2.3. Brachytherapy

For the experimental irradiation setup, each culture plate was positioned on two slabs of water—equivalent material (PMMA/Plexiglas) each measuring 30 × 30 cm with a thickness of 1 cm—to simulate photon scattering and absorption corresponding to human tissue beneath the cell layer. An applicator (VT Vaginal Tube CC, CYL 20 mm CC, Elekta AB, Stockholm, Sweden) was placed at the center of each plate and rigidly fixed in position. The wells contained a fluid depth of 10 mm. A 30 × 30 cm bolus with a thickness of 0.5 cm was positioned above the cells and served as a surface tissue-equivalent material, providing a more clinically realistic dose distribution. To enable accurate geometric reconstruction of the irradiation setup and dosimetric calculation, computed tomography (CT) imaging of the experimental configuration was performed using 120 kVp and 30 mA acquisition parameters, with a slice thickness of 2 mm. The CT images were imported into the Oncentra Brachy treatment planning system (Elekta AB, Stockholm, Sweden), where a treatment plan was generated based on the geometry of the culture plates and applicator positioning. Source dwell positions and dwell times were optimized to achieve uniform dose distribution across all samples. For the central source position, the source-to-cell distance varied between 24.3 and 31.0 mm, depending on the well location within the irradiation field.

**Figure 1:**
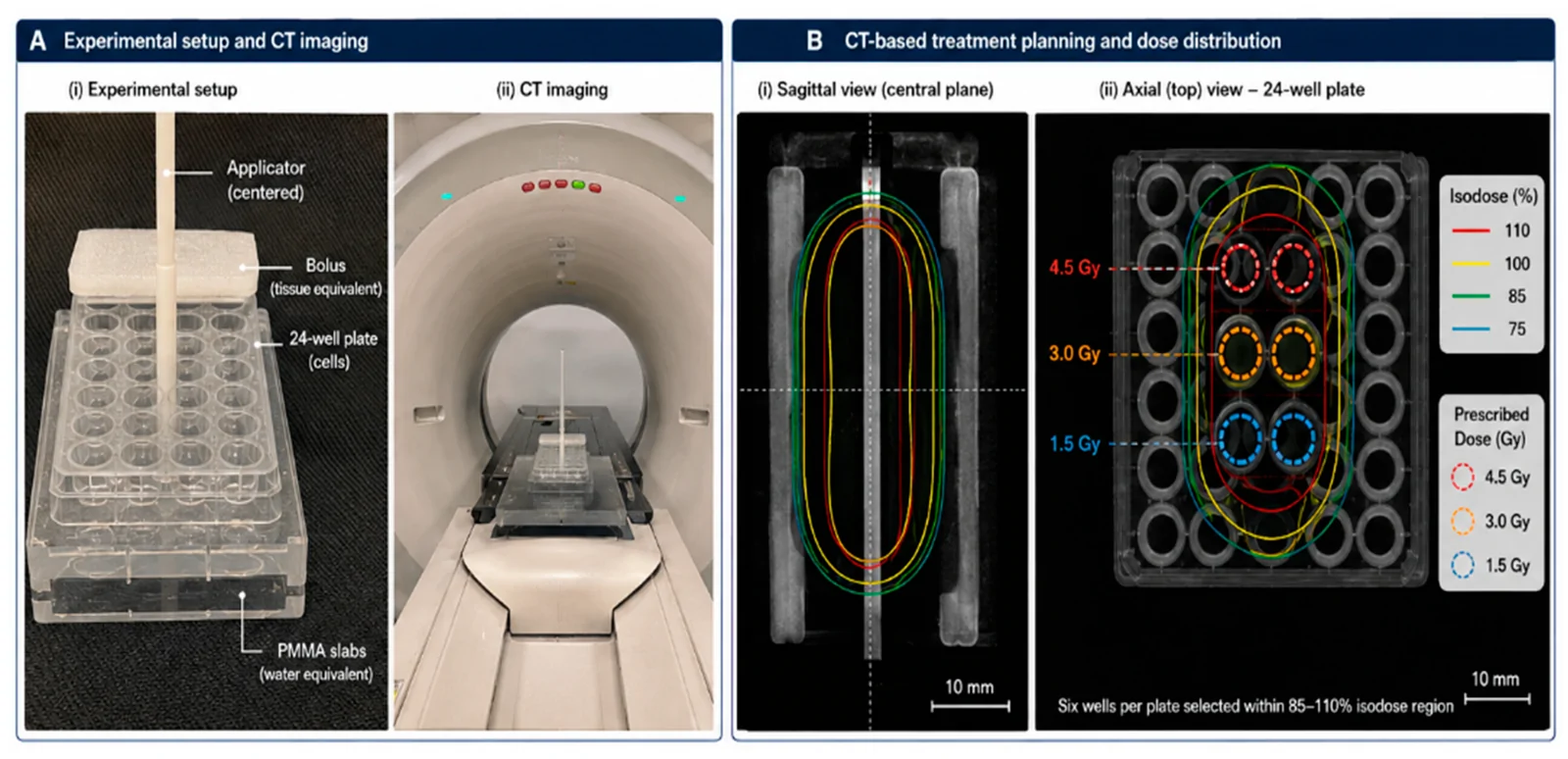
Experimental HDR brachytherapy setup and CT-based dose distribution. (**A**) Experimental irradiation configuration showing the 24-well culture plate positioned between PMMA tissue-equivalent slabs, with a centrally placed applicator and overlying bolus, together with CT imaging of the setup for treatment planning. (**B**) CT-based reconstruction and dosimetric planning performed in the Oncentra Brachy treatment planning system. Isodose distributions are shown in sagittal and axial views. Six wells per plate (dashed circles) were selected within the 85–110% isodose region to receive the prescribed doses of 1.5, 3.0, and 4.5 Gy delivered using an HDR ^192^Ir source.

Brachytherapy irradiation was performed using an Elekta Flexitron HDR afterloading system equipped with an ^192^Ir source. At the time of irradiation, the source air-kerma strength was 19,402.57 cGy·cm^2^/h. Based on the resulting isodose curves, six wells per plate were selected within regions intersected by the isodose lines, ensuring that each well received 85–110% of the prescribed doses of 1.5 Gy, 3 Gy and 4.5 Gy. The inherent dose gradient associated with the ^192^Ir source resulted in variation in the calculated dose across individual wells within this selected isodose region. Dose distribution was therefore assessed based on the CT-derived treatment-planning calculations. Independent experimental dosimetry was not performed, as placement of a dosimeter within the wells was not feasible without altering the experimental geometry. This selection allowed for reliable assessment of cellular response to irradiation. The localization of viable cells at the bottom of the wells was taken into account during dose planning and isodose evaluation. Brachytherapy irradiation was performed using an Elekta Flexitron HDR afterloading system equipped with an ^192^Ir source. Cell samples were irradiated according to the predefined treatment plan, with continuous monitoring of dosimetric parameters and adherence to clinical radiation protection standards. Following irradiation, samples were collected and transferred for subsequent cytological and biological analyses according to the study protocol. The experimental HDR brachytherapy setup and CT-based treatment planning are presented in Figure 1. CT-derived dosimetric calculations were used to optimize source dwell positions and select six wells per plate within the 85–110% isodose region for subsequent biological analysis. For each experimental irradiation, SiHa cells were allocated to three experimental groups: irradiation alone (SiHa), representing the conventional brachytherapy condition, and irradiation following incubation with either 10 nm or 50 nm AuNPs, representing the combined AuNP–brachytherapy treatment conditions. Within the six-well irradiation configuration, two wells were assigned to each experimental group. The complete experimental series was independently performed twice using independently prepared cell cultures, constituting two independent biological replicates (*n* = 2). Each experimental group was evaluated at radiation doses of 1.5, 3.0, and 4.5 Gy, with samples allocated to three predefined post-irradiation assessment time points (0, 24, and 48 h). Samples corresponding to different radiation doses and post-irradiation time points represented distinct experimental conditions and were therefore not considered additional independent biological replicates.

### 2.4. Cell Viability and Apoptosis Assay

Cell viability and apoptosis were assessed using the Dead Cell Apoptosis Kit with Annexin V (Invitrogen, Thermo Fisher Scientific, Waltham, MA, USA). FITC-conjugated Annexin V was used to detect early apoptotic cells, while propidium iodide (PI) was used to identify late apoptotic or necrotic cells. Annexin-binding buffer was used according to the manufacturer’s instructions. Following treatment, cell suspensions were centrifuged at 400× *g* for 5 min, and the supernatant was discarded. Cells were resuspended in Annexin V–PI staining solution and incubated for 15 min at room temperature in the dark. Binding buffer was then added, and samples were gently mixed. Samples were analyzed immediately using an Omnicyt flow cytometer (Cytognos, Salamanca, Spain). Cell populations were classified as viable, early apoptotic, late apoptotic, or necrotic based on Annexin V and PI fluorescence.

### 2.5. Clonogenic Survival Assay and Data Processing

Clonogenic assays were conducted to investigate the effect of ionizing radiation on the long-term survival of cervical cancer cells both in the presence and absence of gold nanoparticles (AuNPs). Cells were exposed to graded radiation doses delivered by an ^192^Ir HDR brachytherapy source and subsequently assessed for colony-forming ability.

Plating efficiency (PE) was defined as the proportion of seeded cells that retained the capacity to form colonies under control conditions. Following irradiation, cell survival was quantified by calculating the surviving fraction (SF), which represents the fraction of cells maintaining clonogenic potential after treatment, normalized to PE.

$$Plating\;Efficiency\;\left(PE\right)\;\left(\%\right)=\frac{No.\;of\;Colonies\;formed}{No.\;of\;cells\;seeded}\times 100$$

$$Surviving\;Fraction\;\left(SF\right)\;\left(\%\right)=\frac{No.\;of\;Colonies\;formed\text{ after }treatment}{No.\;of\;cells\;seeded\times PE}\times 100$$

Radiation-induced cell survival was further analyzed using the linear–quadratic (LQ) model, which describes the relationship between absorbed dose and biological response. The model expresses SF as an exponential function of dose, incorporating a linear component (*α*) associated with damage induced at low doses and a quadratic component (*β*) reflecting increased sensitivity at higher doses.

$$SF=e^{-aD-\beta D^{2}}$$

Survival curves were fitted using a custom MATLAB-based analysis pipeline (MATLAB R2022b, MathWorks, Natick, MA, USA), enabling estimation of *α* and *β* parameters and visualization of dose–response relationships at different post-irradiation time points. The same computational framework was employed to determine the dose enhancement factor (DEF), which quantifies the extent of AuNP-mediated radiosensitization by comparing the radiation doses required to elicit equivalent biological effects in treated and untreated cells.

Additionally, MATLAB-based analysis was applied to visualize apoptotic responses as a function of radiation dose and time after exposure, allowing integration of radiobiological outcomes with observed cellular responses.

### 2.6. Dose Enhancement Factor (DEF)

The radiosensitizing effect of AuNPs was evaluated by calculating the dose enhancement factor (DEF), defined as the ratio of the radiation dose required to achieve an equivalent clonogenic survival in untreated control cells to that required in AuNP-treated cells [30,31]:

$$DEF=\frac{radiation\;dose\;absorbed\;by\;tumor\;cells\;in\;the\;presence\;of\;AuNPs}{radiation\;dose\;absorbed\;by\;tumor\;cells\;without\;AuNPs}$$

The radiation doses in the presence and absence of AuNPs were obtained from the corresponding fitted dose–response survival curves. DEF was determined separately for 10 nm and 50 nm AuNPs at 0, 24, and 48 h post-irradiation over the investigated dose range of 1.5–4.5 Gy. A DEF > 1 indicates enhanced radiosensitivity in AuNP-treated cells. Variability was expressed as standard deviation (SD). Differences between post-irradiation time points were interpreted as changes in the biological radiosensitizing response rather than changes in the physical radiation dose delivered by the HDR ^192^Ir source.

### 2.7. Statistical Analysis

All experiments were performed in two independent biological replicates (*n* = 2). Data are presented as mean ± standard deviation (SD), and error bars represent SD. Given the sample size typical for in vitro radiobiological studies and the inability to assume normal distribution, non-parametric statistical tests were applied. To evaluate overall differences among treatment groups (SiHa cells alone, SiHa + 10 nm AuNPs, and SiHa + 50 nm AuNPs) at each radiation dose (1.5, 3.0, and 4.5 Gy) and time point (0, 24, and 48 h), the Kruskal–Wallis test was performed. This test assessed whether at least one group differed significantly from the others. To examine ordered dose–response relationships within each treatment group, the Jonckheere–Terpstra trend test was used. A decreasing trend was hypothesized for survival fraction, whereas an increasing trend was hypothesized for apoptosis and dose enhancement factor (DEF). Statistical significance was defined as *p* < 0.05.

**Figure 2:**
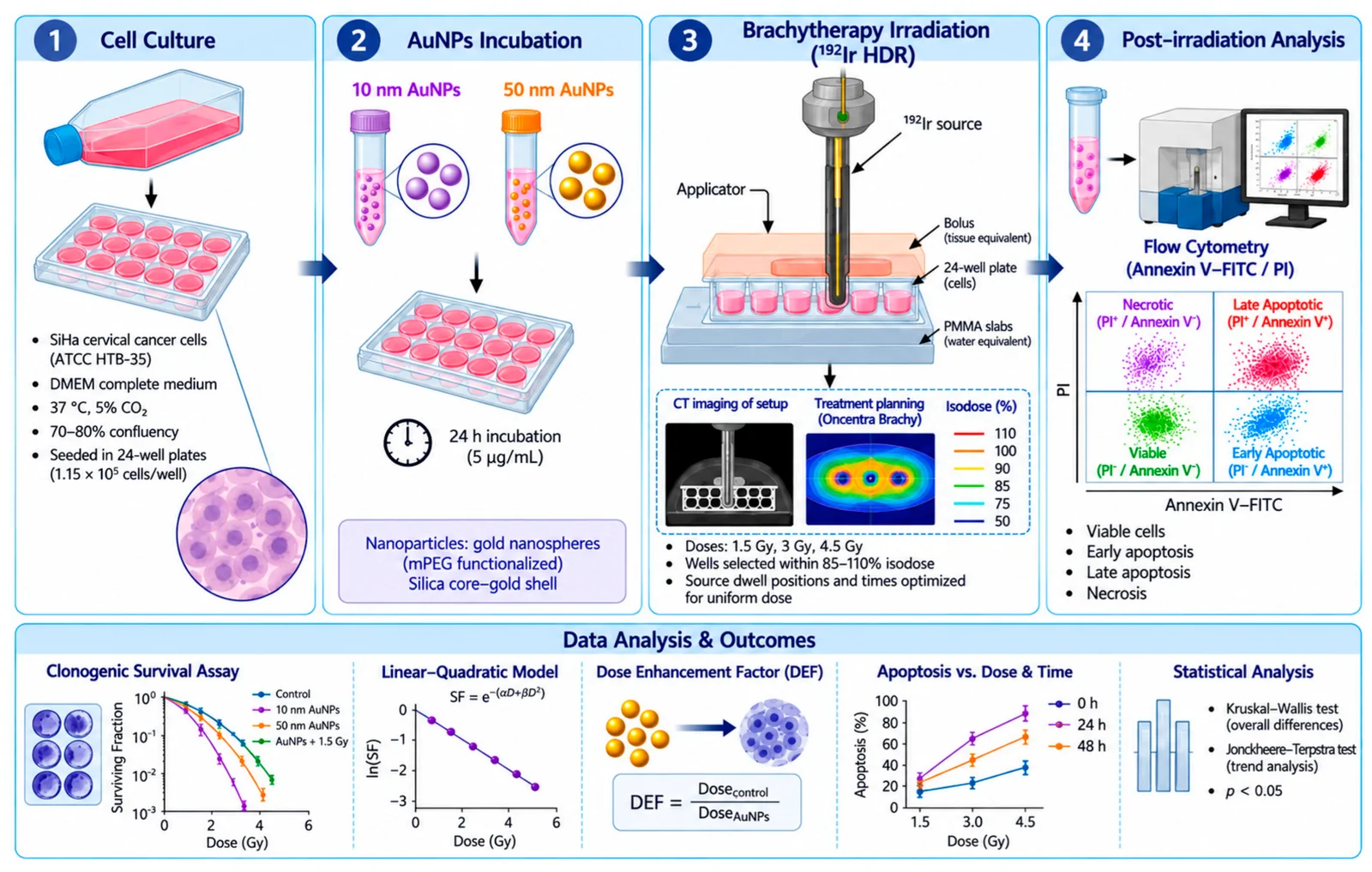
Experimental workflow of the study. SiHa cervical cancer cells were cultured and seeded in 24-well plates, followed by incubation with 10 nm or 50 nm gold nanoparticles (AuNPs) at 5 μg/mL for 24 h prior to HDR ^192^Ir brachytherapy irradiation. Following treatment, cells were collected immediately, at 24 and 48 h post-irradiation for Annexin V/PI flow cytometric analysis. The flow cytometry-derived survival fraction (SF) values were subsequently used for radiation dose–response analysis and Linear–Quadratic (LQ) model fitting, while dose enhancement factor (DEF) values were calculated to evaluate the radiosensitizing effect of AuNP treatment. The complete experimental workflow, from cell preparation and AuNP exposure through irradiation, post-irradiation assessment, and data analysis, is illustrated in the figure.

The experimental design and analytical workflow of the study are summarized in Figure 2. SiHa cervical cancer cells were incubated with gold nanoparticles of different sizes prior to HDR ^192^Ir brachytherapy irradiation, followed by evaluation of clonogenic survival, dose enhancement, and apoptosis at different post-irradiation time points.

## 3. Results

### 3.1. Cell Survival Curves

To accurately assess cell death induced by ionizing radiation, the clonogenic assay was used to evaluate the capacity of cells to retain long-term proliferative potential following irradiation. By analyzing colony survival as a function of absorbed dose, radiation response and treatment efficacy were quantitatively determined. This approach also enabled investigation of radiation response enhancement following intracellular accumulation of gold nanoparticles (AuNPs).

**Figure 3:**
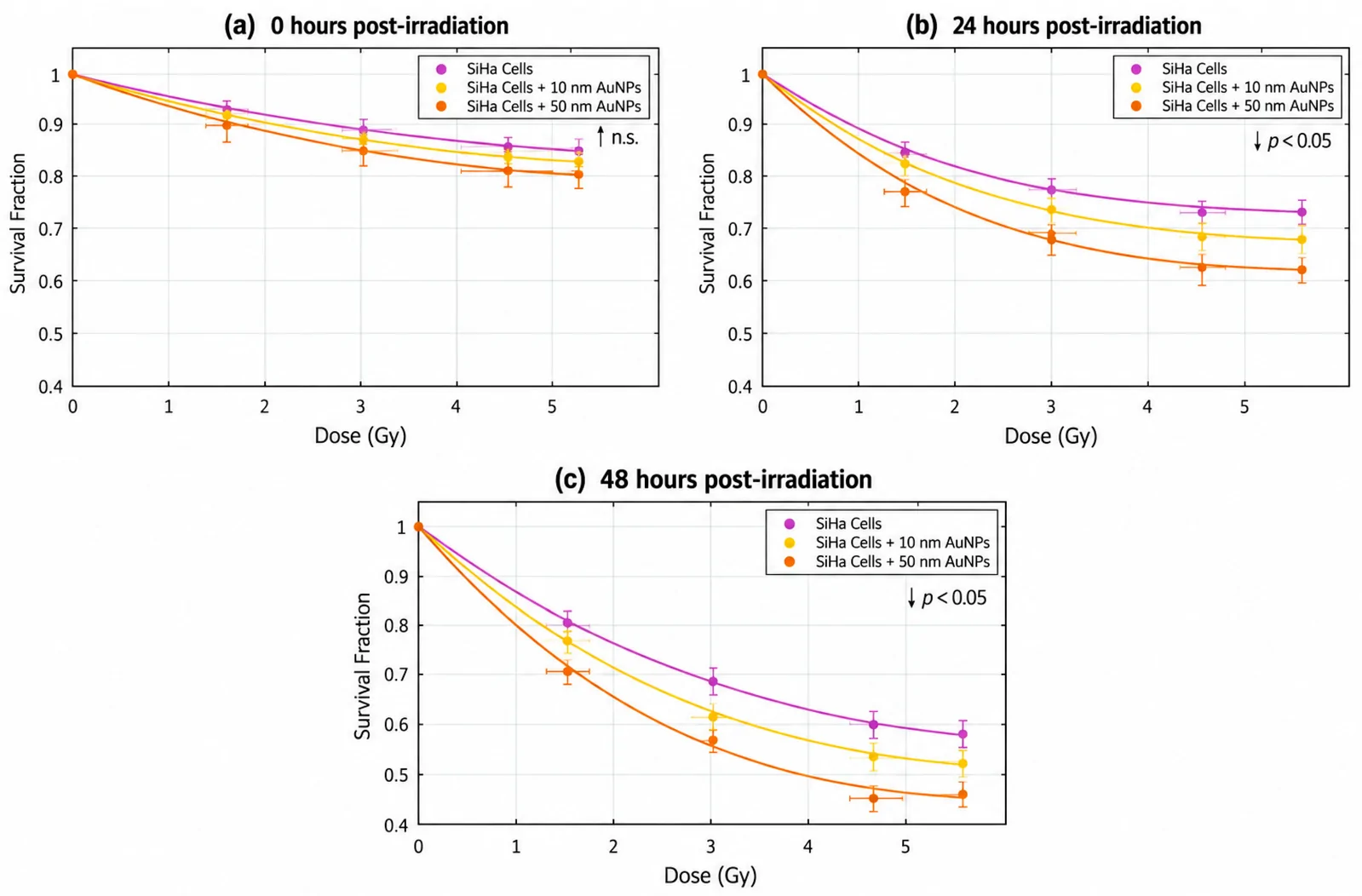
Radiation responses of cells and cells with 5 μg/mL of 10 nm and 50 nm gold nanoshells incubated for 24 h fitted with the linear quadratic model for different post−irradiation times and different deposited doses. Survival fractions over dose of SiHa, with 10 nm (yellow), 50 nm (orange) and without AuNPs (magenta) are depicted for (**a**) 0 h after irradiation. (**b**) 24 h after irradiation. (**c**) 48 h after irradiation. Data are presented as mean ± SD from two independent biological experiments (*n* = 2); error bars represent SD. Symbols/curves correspond to control cells (irradiation alone), cells treated with 10 nm AuNPs, and cells treated with 50 nm AuNPs, as indicated in the figure legend.

In vitro dose enhancement experiments were performed using the SiHa cervical cancer cell line undergoing ^192^Ir HDR Brachytherapy. Cells incubated with 5 μg/mL of 10 nm and 50 nm gold nanoshells for 24 h were evaluated at multiple post-irradiation time points and across a range of delivered doses. The resulting survival data are presented as clonogenic survival curves (Figure 3a–c).

The survival curves were fitted using the linear-quadratic (LQ) model to characterize the radiation response of SiHa cells in the presence and absence of AuNPs. The fitted α and β parameters, together with the corresponding α/β ratios, are summarized in Table 1. Differences in the fitted parameters were observed between the control and AuNP-treated groups and across the different post-irradiation time points. These values provide a quantitative description of the dose–response characteristics observed in the respective experimental conditions.

### 3.2. Dose Enhancement Factor

**Figure 4:**
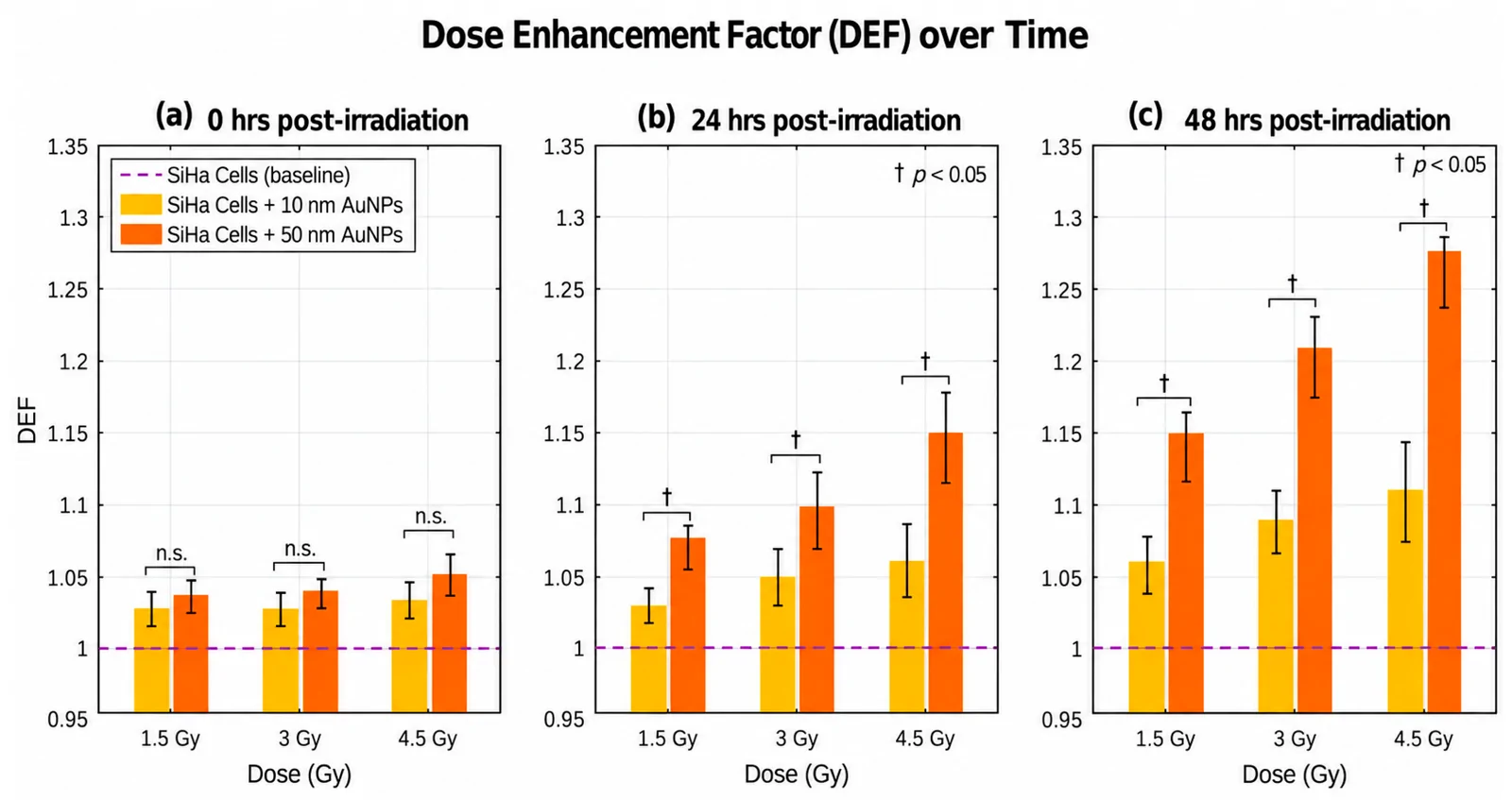
Dose Enhancement Factors for SiHa cells after treatment with different doses and measured for (**a**) 0 h post-irradiation. (**b**) 24 h post-irradiation. (**c**) 48 h post-irradiation. Data are presented as mean ± SD from two independent biological experiments (*n* = 2); error bars represent SD. Symbols/curves correspond to the 10 nm and 50 nm AuNP treatment groups, as indicated in the figure legend. DEF represents the ratio of the radiation dose required to achieve a given biological effect in control cells to that required in AuNP-treated cells. † denotes statistical significance at *p* < 0.05, where applicable.

The outcomes of the clonogenic survival experiments, expressed in terms of the dose enhancement factor across different cell lines, radiation doses, and post-irradiation time points, are presented in Figure 4. These results summarize the impact of gold nanoparticle–mediated radiosensitization on long-term cell survival.

### 3.3. Apoptosis Measurements

#### 3.3.1. Apoptosis over Dose

**Figure 5:**
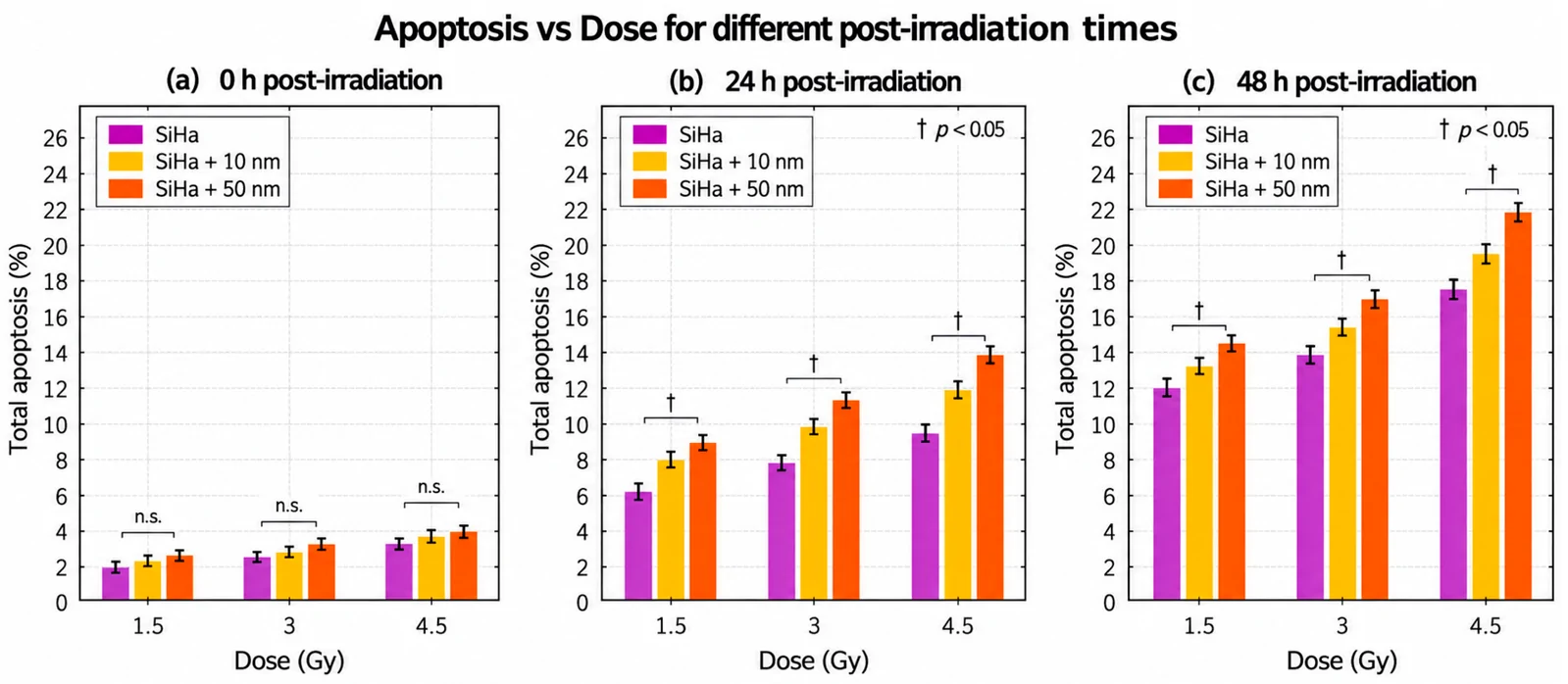
Depiction of apoptosis over dose for cells and cells with 10 nm and 50 nm AuNPs for the doses of 1.5 Gy, 3 Gy, 4.5 Gy for (**a**) 0 h post-irradiation. (**b**) 24 h post-irradiation. (**c**) 48 h post-irradiation. Data are presented as mean ± SD from two independent biological experiments (*n* = 2); error bars represent SD. Symbols/curves correspond to control cells (irradiation alone), cells treated with 10 nm AuNPs, and cells treated with 50 nm AuNPs, as indicated in the figure legend. † denotes a statistically significant difference compared with the corresponding irradiated control (*p* < 0.05).

In parallel with clonogenic survival, apoptotic cell death was evaluated to further characterize radiation-induced biological effects in the presence and absence of gold nanoparticles. Apoptosis was quantified at multiple post-irradiation time points and dose levels to assess the contribution of programmed cell death following exposure to ionizing radiation. The temporal evolution of apoptosis in the SiHa cervical cancer cell line with 10 nm, 50 nm AuNPs and without AuNPs over each dose is shown in Figure 5.

#### 3.3.2. Apoptosis over Time

**Figure 6:**
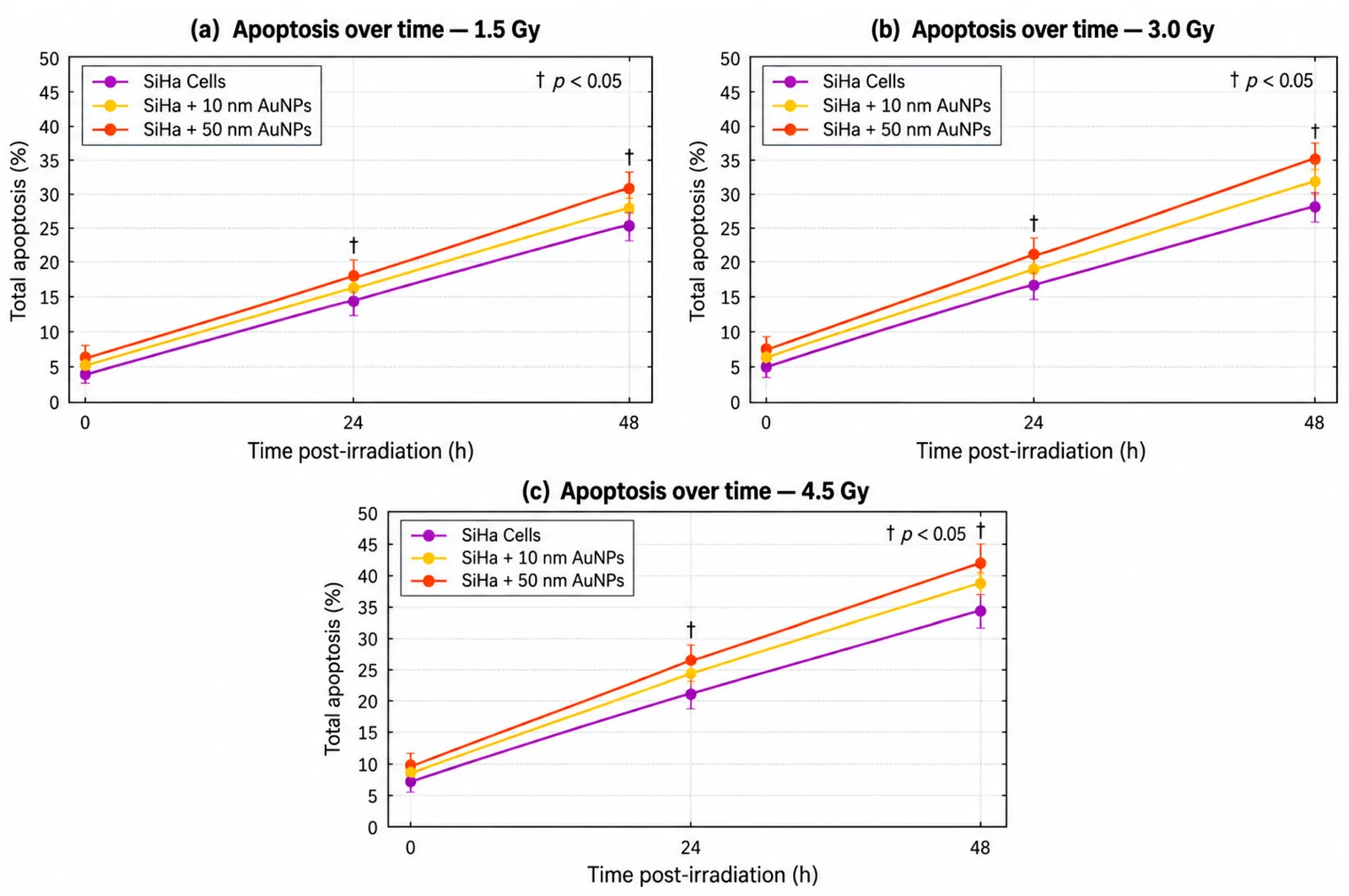
Depiction of apoptosis over time for cells and cells with 10 nm and 50 nm AuNPs over the 0–48 h post irradiation period for (**a**) 1.5 Gy. (**b**) 3 Gy. (**c**) 4.5 Gy. Data are presented as mean ± SD from two independent biological experiments (*n* = 2); error bars represent SD. Symbols/curves correspond to control cells, cells treated with 10 nm AuNPs, and cells treated with 50 nm AuNPs, as indicated in the figure legend. T0, T24, and T48 correspond to measurements immediately after irradiation and at 24 and 48 h post-irradiation, respectively. † denotes a statistically significant difference compared with the corresponding control at the same time point (*p* < 0.05).

Additionally, the relationship between apoptosis and time was evaluated for the three radiation dose levels used. The corresponding results are presented in Figure 6.

## 4. Discussion

The present study investigated the effects of 10 nm and 50 nm gold nanoparticles (AuNPs) on the response of SiHa cervical cancer cells to HDR ^192^Ir brachytherapy. Overall, the results demonstrated a dose- and time-dependent reduction in clonogenic survival following irradiation, with enhanced radiosensitivity observed in the presence of AuNPs. The 50 nm AuNP formulation produced the most pronounced radiosensitizing effect, as reflected by the highest Dose Enhancement Factor (DEF), while also being associated with increased apoptotic cell death. The cellular response varied according to the post-irradiation time point, with the most pronounced effects observed at 24 h. These findings suggest that nanoparticle size and post-irradiation time are important factors influencing the cellular response to HDR brachytherapy in the SiHa model.

### 4.1. Assessment of the Cell Survival Curves

The clonogenic survival curves derived from linear–quadratic (LQ) modelling demonstrate a clear dose-dependent decrease in survival fraction across all experimental groups, with enhanced radiosensitivity in the presence of AuNPs. This observation is consistent with previous studies identifying gold nanoparticles as effective radiosensitizers in vitro [21,32].

This effect is reflected primarily in an increase in the linear component α, which is commonly associated with enhanced induction of irreparable DNA damage, and in some cases a concurrent increase in β, suggesting an additional contribution from sublethal damage accumulation and/or misrepair [33,34]. In the present study, these changes were accompanied by a reduction in the α/β ratio, suggesting a shift toward more complex and less readily repairable damage profiles.

Notably, radiosensitization was greater with 50 nm than with 10 nm AuNPs, demonstrating a size-dependent biological response under the present experimental conditions. Previous experimental studies provide a mechanistic basis for this observation: AuNP size influences membrane interaction and receptor-mediated endocytosis, with intermediate particle sizes around 50 nm frequently showing particularly efficient cellular internalization. Chithrani et al. reported maximal uptake for 50 nm AuNPs compared with smaller and larger particles and subsequently demonstrated that this size was also associated with greater radiation enhancement. Following internalization, AuNPs are commonly trafficked through endosomal–lysosomal compartments, where intracellular clustering may occur. Thus, particle size may influence radiosensitization not only through the amount of intracellular gold, but also through its retention, trafficking, aggregation state, and proximity to radiosensitive cellular structures. In the present study, intracellular gold content and subcellular localization were not directly quantified by elemental analysis or visualized by TEM; therefore, these mechanisms cannot be assigned directly to the greater response observed with 50 nm AuNPs. Rather, our clonogenic and apoptosis results demonstrate the downstream radiobiological consequence of nanoparticle size, while the underlying intracellular mechanisms remain supported by previous experimental evidence [35,36].

Under ^192^Ir brachytherapy conditions, AuNPs are understood to enhance secondary electron emission and reactive oxygen species (ROS) generation, leading to amplified nanoscale energy deposition and enhanced biological damage [10,37]. Collectively, the observed reduction in survival fraction reflects the combined contribution of physical dose enhancement and sustained biological effects, including increased DNA double-strand break formation, elevated oxidative stress, and impaired DNA repair, ultimately resulting in loss of clonogenic capacity [21,38].

### 4.2. Assessment of the Dose Enhancement

According to the results depicted in Figure 4, the dose enhancement factor (DEF) analysis further confirms the radiosensitizing effect of gold nanoparticles, demonstrating a clear dependence on both nanoparticle size and radiation dose. While DEF values remain close to unity under baseline conditions, indicating negligible immediate enhancement, a progressive increase is observed under conditions that allow for the manifestation of biological damage [17,39].

From a radiophysical perspective, the radiation field associated with ^192^Ir should be considered separately from the secondary-electron processes occurring in the presence of AuNPs. ^192^Ir decay involves both photon and β/electron emissions, while additional secondary electrons are generated locally through photon interactions with gold and the surrounding medium, including photoelectrons and electrons arising from subsequent atomic relaxation processes [40,41]. The relative contribution of these components to local energy deposition depends on source geometry, encapsulation, source-to-target distance, and nanoparticle environment, and their quantitative separation would require dedicated coupled photon–electron radiation-transport modelling. In the present experimental configuration, the encapsulated ^192^Ir source provides both photon and β/electron components of the radiation field, which should be distinguished from the secondary electrons relevant to AuNP-associated nanoscale dose enhancement that are generated locally through photon interactions and subsequent atomic relaxation processes in gold and the surrounding medium [42,43,44]. Previous experimental and modelling studies indicate that the interaction between radiation dose and AuNP-mediated radiosensitization should be considered at the nanoscale where the presence of gold modifies the spatial pattern of energy deposition rather than simply increasing the mean absorbed dose [45,46]. The high-Z-dependent enhancement of photon interactions and the resulting emission of photoelectrons, Auger electrons, and other low-energy secondary electrons can produce steep radial dose gradients around individual nanoparticles and nanoparticle clusters [42,47]. Consequently, increasing radiation dose increases not only the number of primary ionization events but also the frequency of AuNP-associated nanoscale energy-deposition events, generating highly heterogeneous regions of locally amplified radiation damage [18,48]. This spatial heterogeneity is biologically relevant because densely clustered ionizations can generate complex DNA damage comprising combinations of strand breaks, base damage, and closely spaced lesions that are considerably less amenable to accurate enzymatic repair than isolated radiation-induced lesions [49,50]. The resulting radiosensitization therefore cannot be described solely by a macroscopic dose-enhancement factor but rather reflects the translation of highly localized physical energy deposition into increased biological effectiveness at the cellular level [21]. An additional contribution arises from the coupling of these physical events with radiation-induced water radiolysis and oxidative chemistry [51]. Short-range secondary electrons generated in the vicinity of AuNPs can increase the local production of reactive radical species, extending the initial physical interaction into a physicochemical phase of molecular injury [13,21,52].

As radiation dose increases, the combined burden of directly induced and ROS-mediated lesions may progressively exceed cellular DNA damage-recognition and repair capacity, increasing the probability of persistent or misrepaired DNA damage. Such unrecoverable damage provides a mechanistic link between nanoscale AuNP-associated dose amplification and the subsequent activation of cellular death responses. In the present experiments, this transition is reflected by the dose-dependent reduction in clonogenic survival together with the progressive increase in apoptosis, particularly in AuNP-treated cells. Thus, the interaction between radiation dose and AuNPs can be interpreted as a multiscale process in which nanoscale physical dose heterogeneity is translated through physicochemical amplification and limitations in damage repair into an enhanced radiobiological response.

Notably, DEF values rise consistently with increasing dose, reaching approximately 1.10–1.15 for 10 nm AuNPs and up to ~1.25–1.30 for 50 nm AuNPs at the highest dose levels, highlighting a pronounced size-dependent effect. This observation is consistent with the findings of Behrouzkia et al., who reported maximal dose enhancement for intermediate-sized AuNPs due to an optimal balance between increased interaction cross section and reduced self-absorption of secondary electrons within the nanoparticle. As nanoparticle size increases, the probability of photon interaction and secondary electron generation also increases; however, excessively large nanoparticles may partially reabsorb emitted electrons within their own volume, limiting electron escape into the surrounding medium. Such effects may facilitate greater escape of photoelectrons and Auger electrons into the surrounding medium, thereby amplifying localized energy deposition and radiosensitization. The size effect is further influenced by the spatial nature of AuNP-mediated energy deposition. Radiation interactions with gold generate short-range secondary electrons and promote local radiolytic and oxidative processes; consequently, biological effectiveness depends not only on the amount of intracellular gold but also on where nanoparticles or nanoparticle clusters are located relative to critical cellular targets [9,45,53]. Monte Carlo studies have shown that variations in AuNP size, clustering, and distance from the nucleus can substantially alter nanoscale energy deposition, reactive-species yield, nuclear dose, and predicted cell survival [43,54]. Therefore, the greater DEF observed for 50 nm AuNPs in the present study should be interpreted as the integrated result of particle-dependent cellular processing and nanoscale radiation interactions, rather than as evidence of increased uptake alone [19,55].

Furthermore, the increasing DEF with dose suggests that AuNP-mediated radiosensitization may be more effective under conditions of higher radiation burden, potentially reflecting cumulative damage processes and reduced repair capacity. Collectively, the DEF results support the conclusion that AuNPs act as effective radiosensitizers, with greater enhancement observed for 50 nm particles and at higher radiation doses.

### 4.3. Assessment of Apoptosis

As depicted in Figure 5, apoptosis shows a clear dependence on radiation dose, increasing steadily as dose escalates across all experimental groups. Higher doses result in a greater burden of DNA damage, exceeding cellular repair capacity and triggering programmed cell death. This effect is further amplified in the presence of AuNPs, particularly for 50 nm particles, which consistently produce the highest apoptotic fractions [56]. Collectively, these findings highlight the critical role of nanoparticle size and radiation dose in modulating apoptotic responses under ^192^Ir brachytherapy conditions [57]. The consistently greater apoptotic response observed with 50 nm AuNPs further supports a size-dependent downstream biological effect. Importantly, this response cannot be attributed to cellular uptake alone [58,59,60]. Size-dependent differences in internalization, intracellular trafficking, retention, and clustering may alter the spatial distribution of secondary-electron energy deposition and ROS generation relative to critical cellular targets, thereby influencing DNA damage signaling, mitochondrial stress, and subsequent activation of apoptotic pathways [61]. Since intracellular AuNP content, subcellular localization, and these intermediate mechanisms were not directly assessed in the present study, apoptosis should be interpreted as an experimentally demonstrated radiobiological endpoint of the size-dependent response rather than evidence of a specific intracellular mechanism [59].

The enhanced apoptotic response observed in AuNP-treated cells can therefore be interpreted as a downstream biological consequence of the amplified and increasingly complex radiation-induced damage described above. As radiation dose increases, the accumulation of persistent DNA lesions and oxidative injury increases the probability that cellular damage exceeds the capacity for successful repair and survival. Under these conditions, the cellular response shifts from damage recognition and attempted repair toward the activation of programmed cell-death mechanisms. The higher apoptotic fractions observed in the presence of AuNPs, particularly at increasing radiation doses, are consistent with this transition and provide a biological correlate of the enhanced radiation response demonstrated by the clonogenic assay. Importantly, apoptosis in the present study should be considered an experimentally measured downstream endpoint of radiosensitization rather than evidence for activation of a specific molecular apoptotic pathway, since individual signalling components were not directly investigated.

In parallel, the temporal analysis of apoptosis, as depicted in Figure 6, demonstrates that radiation-induced cell death evolves progressively following irradiation, consistent with the delayed nature of biologically mediated responses. Rather than occurring immediately, apoptosis reflects the outcome of ongoing cellular processes, including DNA damage recognition, repair attempts, and subsequent activation of cell death pathways when damage proves irreparable [62]. The presence of gold nanoparticles (AuNPs) enhances this response, with treated cells consistently exhibiting higher apoptotic levels compared to controls. Among the tested conditions, 50 nm AuNPs induce the most pronounced effect, supporting a size-dependent radiosensitization likely driven by increased intracellular accumulation and sustained oxidative stress [63]. This prolonged biological impact is consistent with enhanced production of reactive oxygen species (ROS) and disruption of mitochondrial function, ultimately promoting apoptotic signaling [57]. The persistence of a stronger apoptotic response in the 50 nm group therefore suggests that AuNP-mediated radiosensitization extends beyond the initial physical radiation interaction and is reflected in the subsequent evolution of the cellular response [45,64,65,66].

### 4.4. Interpretation of Statistical Findings

The use of non-parametric testing ensured robust evaluation of treatment effects without assuming normal distribution of the data.

No statistically significant differences were observed immediately after irradiation (0 h), indicating that radiation-induced biological effects had not yet manifested at this early time point. However, significant overall differences among treatment groups were detected at 24 h and 48 h for survival fraction and apoptosis, as demonstrated by the Kruskal–Wallis analysis. These findings indicate that the presence of gold nanoparticles significantly alters cellular response to brachytherapy over time.

Furthermore, the Jonckheere–Terpstra test confirmed significant monotonic dose–response relationships at 24 h and 48 h. A statistically significant decreasing trend in survival fraction and an increasing trend in apoptosis and DEF were observed with increasing radiation dose. These results demonstrate a consistent and biologically coherent dose-dependent radiosensitization effect.

Taken together, the complementary application of Kruskal–Wallis and Jonckheere–Terpstra analyses provides strong statistical support for a time- and dose-dependent enhancement of radiation-induced cytotoxicity by AuNPs, with the 50 nm nanoparticles exhibiting the most pronounced effect.

### 4.5. Limitations

The findings of the present study should be considered within the scope of the experimental model employed. The use of a clinical ^192^Ir HDR brachytherapy source together with clonogenic survival, DEF, and apoptosis assessment provides complementary evidence of AuNP-mediated radiosensitization in SiHa cells. However, the use of a two-dimensional monoculture and the absence of normal cervical cells and dedicated AuNP-only controls limit the extent to which the findings can be generalized to more complex biological systems. Similarly, the mechanistic interpretation of the observed effects would benefit from further characterization of nanoparticle localization, oxidative stress, and DNA damage responses. The limited number of biological replicates (*n* = 2) also supports interpreting the statistical findings as exploratory. Future studies incorporating additional cellular models, three-dimensional systems such as spheroids or organoids, experimentally verified dosimetry, and complementary mechanistic endpoints will help further establish the biological relevance of AuNP-mediated radiosensitization under HDR brachytherapy conditions.

## 5. Conclusions

This study demonstrates that gold nanoparticle–mediated radiosensitization enhances the radiobiological response to high-dose-rate ^192^Ir brachytherapy in vitro, as demonstrated by reduced clonogenic survival, increased DEF, and enhanced apoptotic response under the investigated conditions. While the physical component of enhancement is understood to involve increased photon interactions and the generation of secondary electrons, including Auger electrons, the overall radiobiological response is strongly dependent on biological parameters such as nanoparticle size, intracellular interactions, and post-irradiation cellular dynamics. In particular, 50 nm AuNPs produced greater radiosensitization than 10 nm AuNPs under the investigated conditions, emphasizing the critical role of nanoparticle size in modulating energy deposition and biological response.

Importantly, the findings reveal that the biological response following irradiation continues to evolve over time, with enhanced apoptotic activity observed at later post-irradiation intervals. These temporal effects highlight the importance of investigating radiation-induced biological responses beyond acute exposure. Potential mechanisms involving oxidative stress, DNA damage, mitochondrial dysfunction, and apoptotic signaling may contribute to these effects, as supported by previous studies, but were not directly investigated in the present work. The use of cervical cancer cell lines provides a relevant experimental platform for investigating nanoparticle-assisted brachytherapy under controlled ^192^Ir irradiation conditions.

Overall, the present findings provide preliminary in vitro evidence supporting the potential of AuNPs as radiosensitizing agents in ^192^Ir HDR brachytherapy and show that the observed response varies with nanoparticle size, radiation dose, and post-irradiation time. However, the in vitro design, limited number of independent biological experiments, and absence of direct measurements of AuNP uptake and subcellular localization or of the proposed molecular mechanisms represent important limitations of the present study. Further experimental validation, including increased biological replication, direct characterization of nanoparticle–cell interactions and mechanistic endpoints, and ultimately in vivo investigation, will be required to determine whether the observed effects translate into improved tumor control or normal-tissue protection. Nevertheless, by integrating radiophysical and radiobiological assessment, the present study provides a basis for further investigation and optimization of AuNP-assisted ^192^Ir brachytherapy.

## Figures and Tables

**Table 1:** Fitted α, β, and α/β parameters of SiHa cells treated with ^192^Ir HDR brachytherapy in the absence and presence of 10 nm and 50 nm AuNPs at different post-irradiation time points.

		α (Gy^−1^)	β (Gy^−1^)	αβ
0 h	SiHa cells	0.041	0.003	15.046
	SiHa cells + 10 nm AuNPs	0.049	0.004	13.613
	SiHa Cells + 50 nm AuNPs	0.057	0.004	13.867
24 h	SiHa cells	0.121	0.012	8.971
	SiHa cells + 10 nm AuNPs	0.137	0.013	10.335
	SiHa Cells + 50 nm AuNPs	0.168	0.016	10.598
48 h	SiHa cells	0.183	0.014	12.952
	SiHa cells + 10 nm AuNPs	0.229	0.019	11.811
	SiHa Cells + 50 nm AuNPs	0.285	0.025	11.479

## Data Availability

The data supporting the findings of this study are available from the corresponding author upon reasonable request.
